# Comfortable and dermatological effects of hot spring bathing provide demonstrative insight into improvement in the rough skin of Capybaras

**DOI:** 10.1038/s41598-021-03102-4

**Published:** 2021-12-08

**Authors:** Kengo Inaka, Tohru Kimura

**Affiliations:** Laboratory Animal Science, Joint Faculty of Veterinary Medicine, Yamaguchi University, 1677-1, Yoshida, Yamaguchi, 753-8515 Japan

**Keywords:** Skin diseases, Therapeutics

## Abstract

The purpose of this study was to clarify dermatologically the favorable effects of hot spring bathing on the rough skin in Capybaras. Non-volcanic hot springs used in this study showed alkaline quality of water (pH 9.3), containing sodium and chloride ions. The normal skin in Capybaras was characterized by the presence of relatively thick epidermis with mild alkaline state (pH 8.26). The dorsal skin had melanin granules in the basal layer. Their rough skin affected in the Japanese cold winter was improved by daily bathing in an alkaline hot spring. The skin properties returned to the normal skin conditions (moisture, melanin and erythema values) observed in the summer. The facial expression mainly changes in the eyes was scored to evaluate comfortable status. The comfortable status during hot spring bathing significantly increased as compared with that observed before bathing (p < 0.01). The thermography revealed a heat retention effect of body temperature after hot spring bathing for 30 min. In conclusion, this study demonstrates that hot spring had significantly comfortable and dermatological effects on the basis of evaluation for the skin and body conditions in Capybaras.

## Introduction

Hot spring bathing is widely used for the regulation of human physical conditions. Hot spring bath is pleasant to take and bathing has very few adverse effects during a long-term treatment. In humans, hot springs have been reported to improve skin condition^1,2^, and it is also known that hot spring therapy has especially beneficial effects on dermatology such as atopic dermatitis, ichthyosis and psoriasis^3–7^. In ancient Japanese hot spring villages, there are many traditions that various wild animals found highly efficacious hot springs in wound healing. These therapeutic effects of hot spring bathing on the skin have not been fully elucidated in human　medicine. The reason is that there are few laboratory animals that are readily available to evaluate dermatologically the effects of hot spring bathing.

Capybaras can be seen in zoos and parks in Japan. Zoo-kept Capybaras are gentle and they become very fond of humans. At zoo-attractions, Capybaras have a relaxed expression on their face in the hot spring. Hot spring therapy, balneotherapy and therapeutic thermal baths offer a healthy and relaxing atmosphere to human patients^8–10^. The benefits of relaxation and stress itself should not be underestimated in these patients.

Recently, we have noticed that Capybaras developed rough dry skin in the winter. Capybaras are the biggest rodents in the world, native to South America. Capybaras originally live in the climate of high-temperature and high-humidity, and they spend a lot of time underwater. Capybaras prefer to soak in a hot spring in the cold winter.　Because of their characteristics, we used these animals to evaluate beneficial effects of hot spring bathing on the rough skin.

The purpose of this study was three-fold: first, to clarify the functional properties of hot spring water, second, to investigate the differences between the skin in the winter (rough skin) and in the summer (normal skin), and third, to demonstrate the comfortable and dermatological effects of consecutive hot spring bathing with the rough skin of Capybaras.

## Results

### Hot spring ingredients

The ingredients of Yuda hot spring are shown in Table 1.

**Table 1:** Components analysis of Yuda hot spring water.

Temperature ($^\circ$C)	74.50
pH	9.30
**Components (mg/L)**	
Na^+^	204.00
K^+^	4.10
Mg^2+^	0.00
Ca^2+^	8.10
Sr^2+^	0.10
Fe^2+^, Fe^3+^	0.00
Mn^2+^	0.00
Al^3+^	0.04
Zn^2+^	0.00
Cu_2_^+^	0.01
H^+^	0.00
Li^+^	0.20
F^-^	10.00
Cl^-^	261.30
Br^-^	0.70
I^-^	0.00
HCO_3_^2-^	11.00
CO_3_^-^	19.90
SO_4_^2-^	18.30
HS^-^	2.00
S_2_O_3_^2-^	0.40
OH^-^	0.30
PO_4_^3-^	0.02
BO_2_^-^	4.50
**Non-dissociated component (mg/L)**	
H_2_SiO_3_	85.90
Total dissolved matter	630.80

The main component of the cation was sodium ion, and the main component of the anion was chloride ion. The hot spring water pH indicates alkaline quality of water.

### Skin conditions in the summer and winter

**Figure 1:**
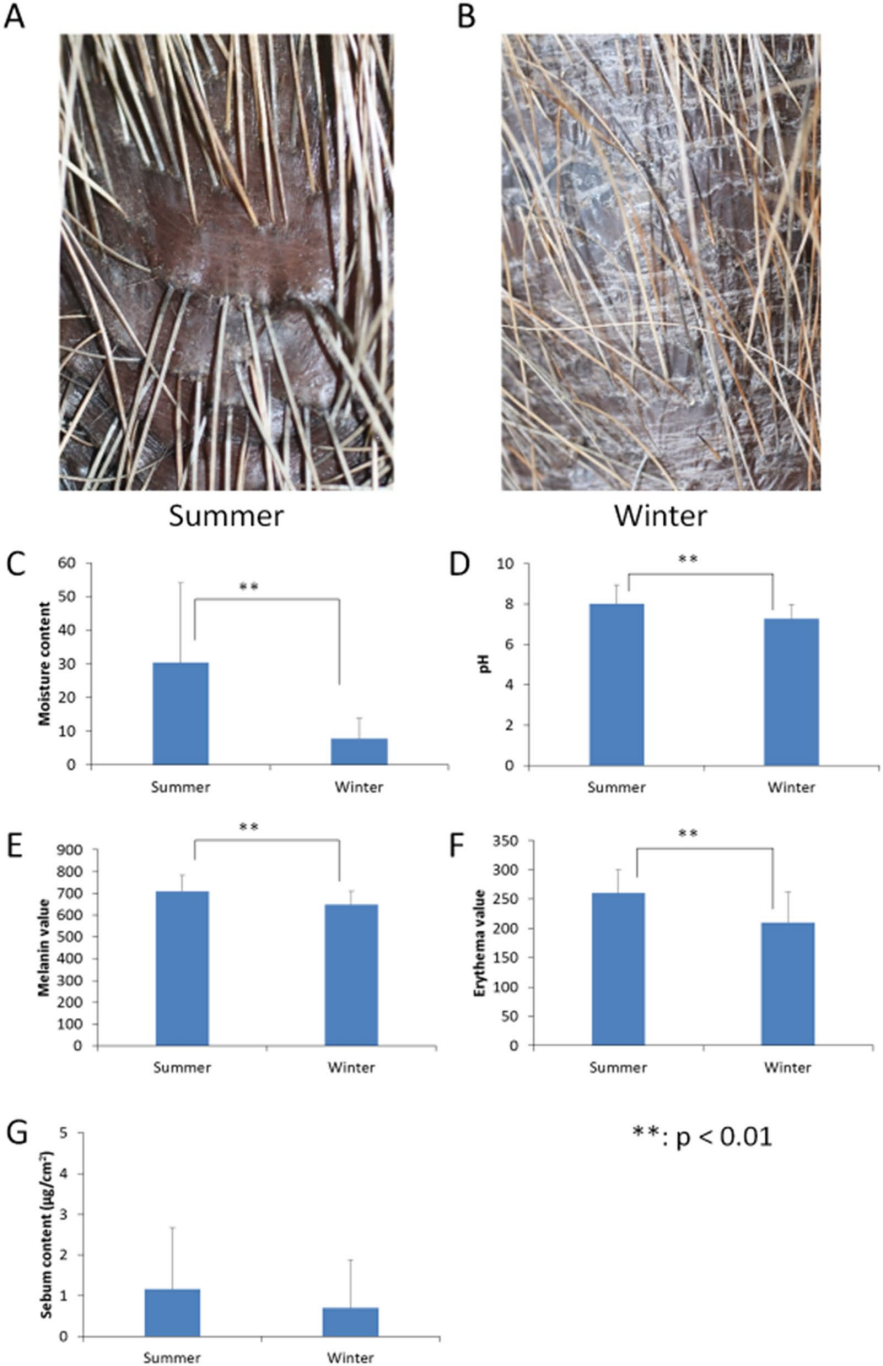
Skin conditions in the summer and winter. (**A**,**B**) Comparison of the summer and winter dorsal skin of Capybaras bred in Japan. The dorsal hair of Capybaras were low density and hard. In summer, the dorsal skin appeared to be reddish black and Capybaras had clear and smooth skin. By contrastin winter, the appearance of the skin deteriorated and the surfaces showed rough skin with crusts and scales. (**C**) The moisture content in winter was almost quarter as much as that in winter. There was significant difference between the moisture content in summer and in winter (p < 0.01). (**D**) The skin pH in winter was significantly lower than that in summer (p < 0.01). In the summer, the skin pH was 8.26, indicating that the skin of Capybaras were naturally weakly alkaline. (**E**) Melanin values in winter were significantly lower than those in summer (p < 0.01). (**F**) Erythema values in summer was significantly higher than those in winter (p < 0.01). (**G**) There was no significant difference of sebum content between in summer and in winter.

Macroscopic findings of the dorsal skin obtained in the summer and winter experiments is shown in Fig. 1A,B. In the summer, the dorsal skin appeared to be reddish black and Capybaras had clear and smooth skin. By contrast, in the winter, the appearance of their skin deteriorated and the surfaces showed rough skin with crusts and scales.

The comparison of the skin properties of Capybaras in the summer and winter is shown in Fig. 1C–G, and all results are shown as mean ± SD. The skin moisture content in the winter (7.85 ± 6.10) was significantly lower than that in the summer (30.55 ± 25.27) (p < 0.01), and in the winter it was reduced by nearly quarter of that in the summer. There was a significant difference between the skin pH in the summer (8.26 ± 0.90) and winter (7.25 ± 0.70) (p < 0.01). The data showed that the normal skin of Capybaras was originally mild alkaline. Skin melanin values in winter (647.27 ± 61.45) were significantly lower than those in summer (707.27 ± 71.77) (p < 0.01). Skin erythema values also showed a similar tendency, and these values were significantly lower in winter (210.33 ± 51.88) than in summer (262.15 ± 41.85) (p < 0.01). There was no significant difference between the skin sebum content determined in both of the seasons. The skin sebum contents were extremely small, ranging from 0 to 2 μg/cm^2^.

### Skin histological examination

**Figure 2:**
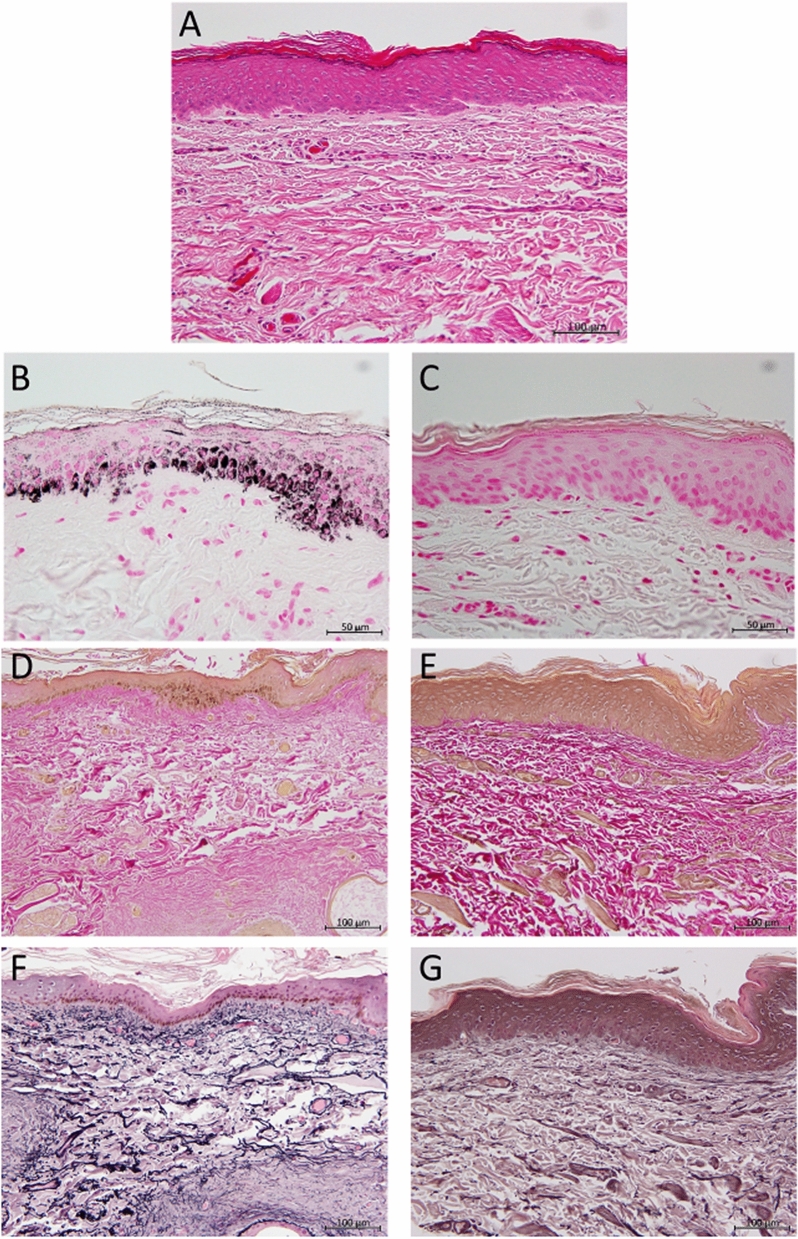
Capybaras skin tissue (**A**) The Capybaras skin had a thick and flat epidermis composed of the keratinous layer, granular layer, prickle layer and basal layer. The dermal papilla was not observed in the border between the epidermis and the dermis. In the dermis, there were no eccrine sweat glands, and only a few sebaceous glands were found in the skin. HE stain. Bar = 100 µm. (**B**) The dorsal epidermis had melanin deposition, and in particular, it was often found in the basal layer. FM stain. Bar = 50 µm. (**C**) The abdominal skin had no melanin granules. FM stain. Bar = 50 µm. (**D**) The collagen fiber density in the dorsal dermis was partly low. WG method. Bar = 100 µm. (**E**) The collagen fibers of the ventral skin were evenly distributed. WG method. Bar = 100 µm. (**F**) The elastic fibers of dorsal skin were thick and proliferated, and the structure was clearly different from that of abdominal skin. vG method. Bar = 100 µm. (**G**) The elastic fibers of the abdominal skin were composed of fine fibers. vG method. Bar = 100 µm.

The histological examination of skin tissue of Capybaras are shown in Fig. 2.Histological findings showed the epidermis was composed of 5–10 layers of epidermal cells, which were clearly divided into four layers: keratinous layer, granular layer, prickle layer and basal layer. The thickness of the epidermis was 40–120 µm. The epidermis and dermal papilla were not developed and were flat, and the dermis was flat and thick. There were no eccrine sweat glands in the dermis, while the small number of sebaceous glands were noted in the skin. In addition, melanin granules were distributed throughout all the dorsal epidermis layers. Melanin deposition, in particular, in the basal layer was remarkable. In contrast, abdominal skin tissue had no melanin granules, though the structure of the epidermal layer was not different from that on the dorsal. In the dermis, the density of collagen fibers was partially low in the dorsal skin. In addition, the elastic fibers of the dorsal skin were thicker and more densely intertwined than those of the abdominal skin. In the upper and lower layers of the dermis of the abdominal skin, fine elastic fibers were observed.

### Hot spring bathing experiment

The hot spring tests were conducted under the cold winter conditions (atmospheric temperature: 4.98 ± 2.10 $^\circ$C). Optimal skin property was measured in the summer (atmospheric temperature: 24.47 ± 2.20 $^\circ$C).

**Figure 3:**
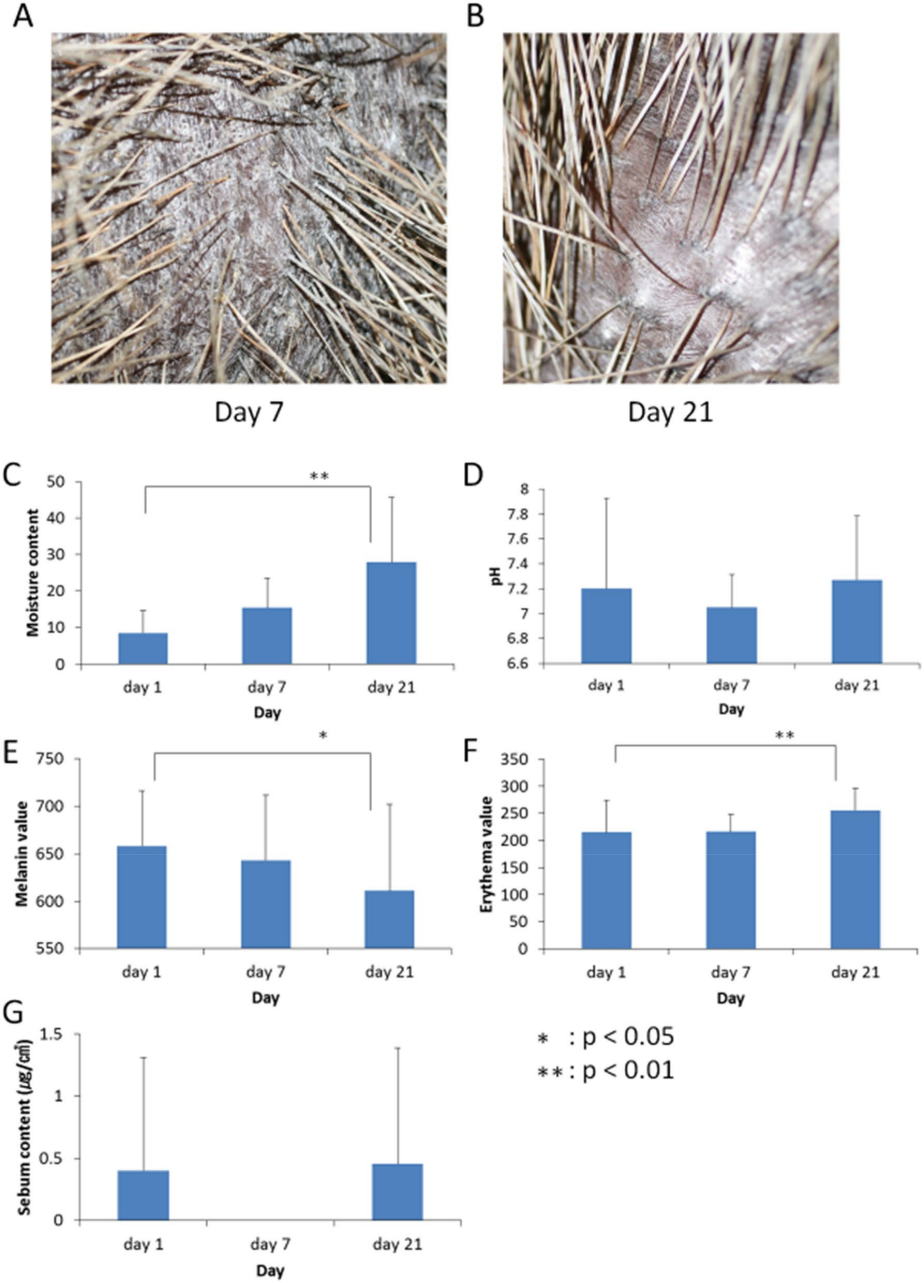
Hot spring bathing experiment. (**A**,**B**) Changes in macroscopic findings of Capybaras skin during hot spring bathing test. On day 7, the skin of Capybaras still contained some apparent scales, and there was no significant change in appearance. On day 21, their skin became smoother compared to scaly rough skin on day 7. The complexion of their skin also appeared to be closer to that of summer. (**C**) The moisture content increased during experiment, with a significant difference between day 1 and day 7. (**D**) The skin pH was neutral throughout the bathing test and not affected by hot spring bathing. (**E**) The melanin values gradually decrease during the test. The values on day 21 were significantly lower than that on day 1. (**F**) The erythema values increased during the bathing test and there was a significant difference between day 1 and day 21. (**G**) The sebum content of Capybaras was always less than 1 µg/cm^2^. No significant changes were seen in the sebum content throughout the test.

The changes of macroscopic findings in the hot spring bathing test are shown in Fig. 3A,B. On day 7, scales were still visible overall, and the condition of the skin seems to be harmed. But their rough skin with scales has become smoother by 21 days after beginning of the hot spring bathing test. The complexion of the skin also changed and looked more like the original reddish-black color. Additionally, the bathing made their skin and hair glossy and the skin texture considerably improved as compared with the skin condition before the hot spring bathing test.

The changes in the dermatological characteristics of Capybaras during hot spring bathing are shown in Fig. 3C–G. The results are shown as mean ± SD. Before beginning of the study, the animals developed apparent dry skin (xerosis cutis) in the midwinter, indicating a marked decrease in skin humidity. Skin moisture chronologically increased during hot spring bathing, and there was a significant difference between day 1 (8.49 ± 6.23) and day 21 (27.86 ± 17.8) (p < 0.01).

The skin pH meter indicated that the skin was neutral condition before beginning of this study. The skin pH levels remained unaffected during a series of the bathing experiments.

The melanin values gradually decreased in process of bathing. The melanin values were significantly declined until day 21 (p < 0.01).

The erythema values ranged from (day 1: 215.0 ± 59.1) to (day 7: 216.2 ± 31.5). The erythema values on day 21 were significantly higher than those obtained on the initial day before bathing (p < 0.01).

Sebum content was less than 1 µg/cm^2^ throughout this test. Little sebum was secreted from the skin of Capybaras.

### Evaluation of comfortable status

**Figure 4:**
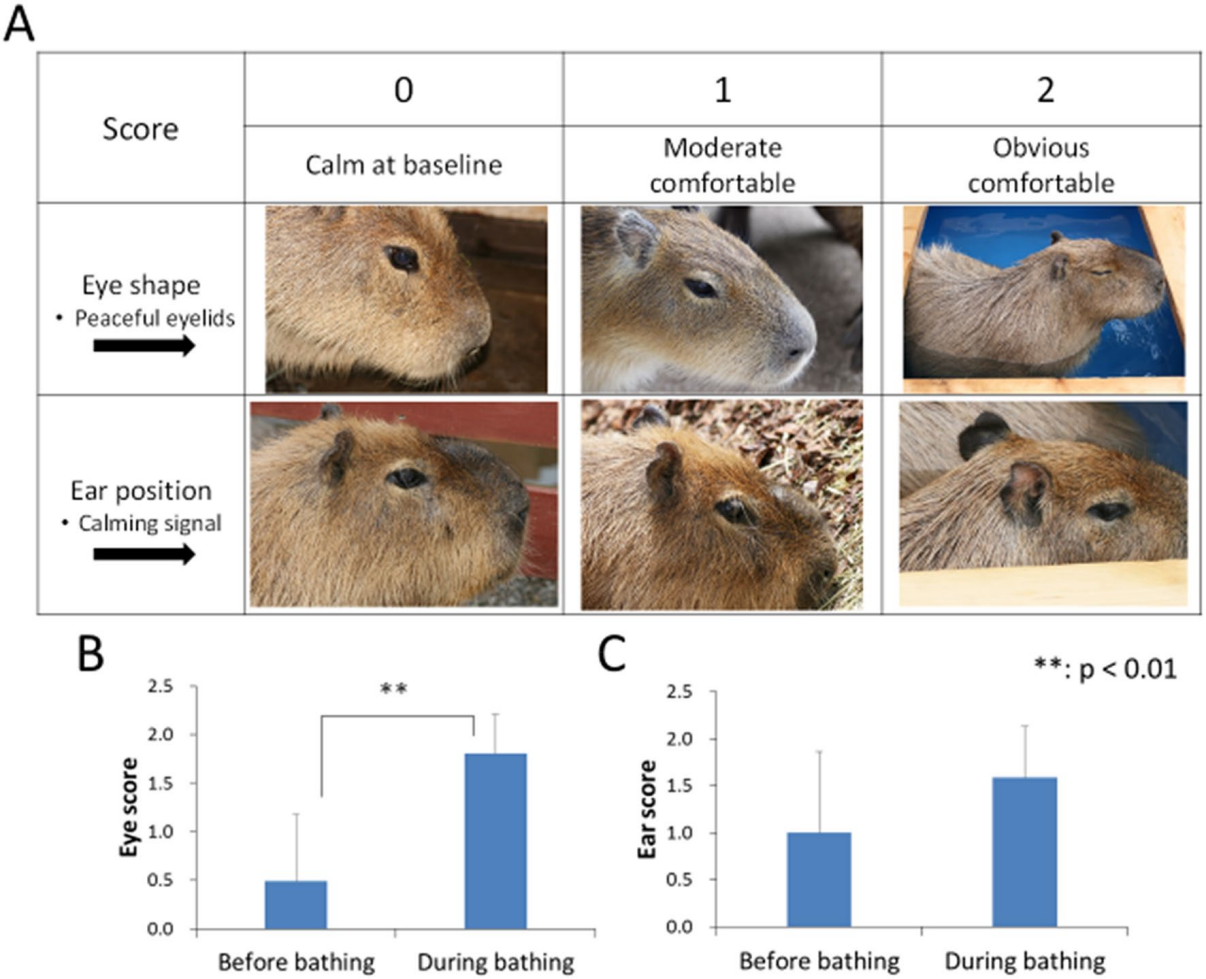
Comparison of facial expression of Capybaras between before bathing and during bathing. (**A**) Scoring table of comfortable status in Capybaras. When the Capybaras feels comfortable, the eyelids close and the visible area of the eyeball becomes smaller, and the ears are gradually pulled back against the body. (**B**) Eye score showed significant difference between before bathing and during bathing (p < 0.01). (**C**) Ear score showed no significant difference before and during bathing.

The results of comfortable status are shown in Fig. 4. The comfortable status were evaluated by the criteria for facial expression. Eye score before bathing (0.49 ± 0.69) was categorized between baseline and moderate comfortable. Eye score during bathing (1.80 ± 0.40) was more than three times as high as that before bathing, and there was a significant difference (p < 0.01). Ear scores before bathing (1.00 ± 0.86) showed moderate comfortable. Ear score during bathing (1.59 ± 0.55) was elevated compared to that before bathing and was classified as moderate to obvious comfortable, but significant difference was not observed (p = 0.19). There was a significant positive correlation between eye score and ear score(p = 0.001), and Spearman's rank correlation coefficient (r_s_) was 0.35.

### Skin thermography

**Figure 5:**
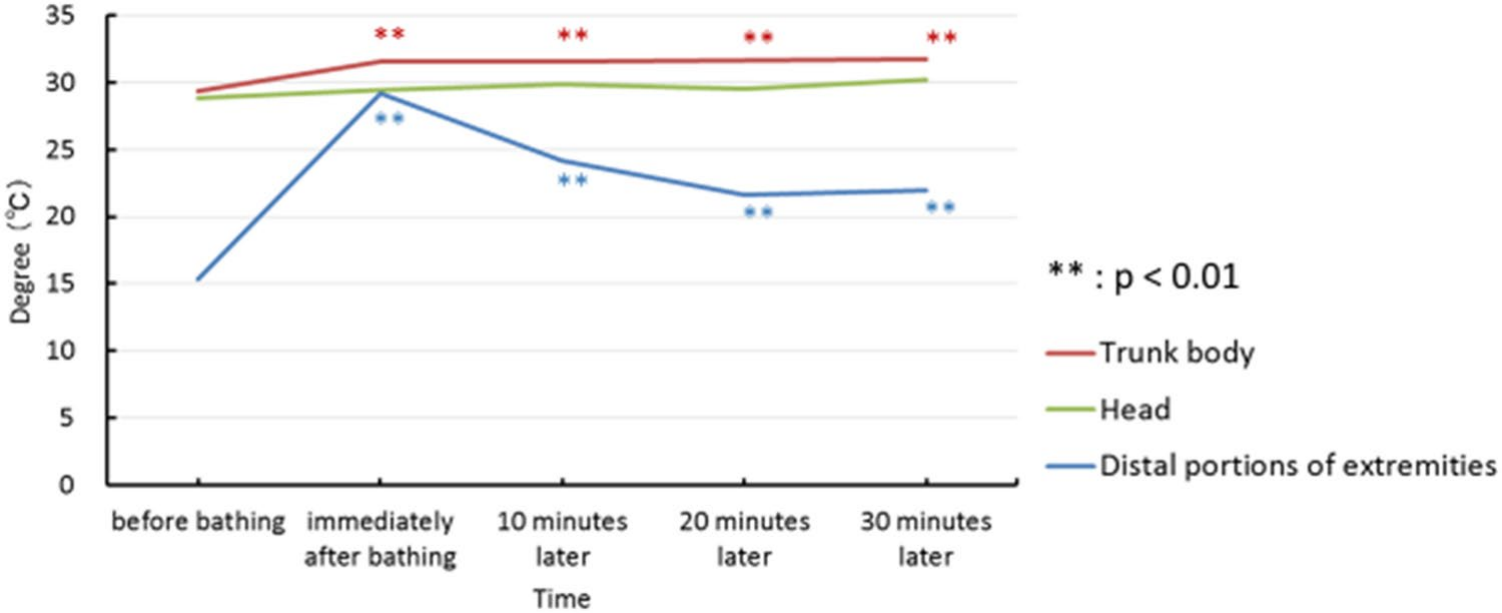
The changes in body surface temperature after hot spring bathing within 30 min following hot spring bathing. Trunk body: Compared to before bathing, the temperature was constantly maintained significantly higher for 30 min. Head: No significant temperature change was observed throughout the experiment. Distal portions of extremities: Although there was a gradual decrease in the temperature after bathing, it continued to maintain a significantly higher temperature than before bathing.

The changes in thermographic data are summarized in Fig. 5. Before bathing, three sites examined in this study were lowered to 28.83 ± 3.38 $^\circ$C: head, 29.34 ± 4.09 $^\circ$C: trunk body, and 15.34 ± 3.87 $^\circ$C: distal portions of extremities in the winter season. The head skin temperature was unaffected during this experiment. No significant difference was statistically found in the temperature during the course of the study. On the trunk body, hot spring bath kept the skin temperature constant at 32 $^\circ$C throughout the experiment, and the skin temperature remained significantly higher than that of before bathing (p < 0.01). Before bathing, the skin temperature of their extremities was about 15 $^\circ$C lower than that of the other sites. Immediately after bathing, the temperature rapidly increased to nearly 30 $^\circ$C. Although the skin temperature gradually decreased after bathing, increased skin temperature was maintained at the level of higher than 30 $^\circ$C for 30 min, in comparison with the initial temperature before bathing (p < 0.01).

## Discussion

Although Yuda hot spring is non-volcanic hot spring, the temperature of the source was very high as compared with the standard value of hot spring temperature. The pH of Yuda hot spring was higher than the average pH of Japanese hot spring^11^. Analysis of the hot spring water showed that the total amount of dissolved components were less than 1000 mg/kg. The hot spring used in this study was classified as simple alkaline water, containing a relatively large amount of sodium and chloride ions. In addition, this hot spring water had a character of low stimulation to the skin. The therapeutic effect appears to lie in some local interactions between hot spring water and the surface structure of the skin.

In this experiment, the skin moisture content was significantly higher in summer than in winter. Zoo-kept Capybaras showed that summer season favored the moist skin and higher moisture content. Previous studies described that the skin moisture in humans was influenced by the turn of the seasons^12,13^. Capybaras naturally live in the tropical marshland of the Amazon Valley with abundant organic matters. Capybaras potentially required no high water retention in the skin, because it was also reported in humans that skin properties about transepidermal water loss and moisture content differ depending on the region and race^12,14^. Their skin was highly susceptible to the changes in environmental temperature and humidity. Winter climate caused apparent rough skin in Capybaras.

The skin pH also depended on the circumstances of seasonal climate. The largest rodent, Capybaras had weakly alkaline feature in the normal skin, while the other rodents (mice, rats and guinea pigs) had weakly acidic skin^15,16^. The skin pH in Capybaras indicated unique features similar to that in companion animals such as cats and dogs^16,17^. Rough skin in the winter showed significantly decreased pH level in association with the reduced moisture content. Also in humans, it has been reported that the skin pH changes when the skin condition deteriorates^18–20^. The present data revealed that both of the parameters were available for indication of the rough skin in Capybaras.

Skin melanin values of Capybaras in summer were significantly higher than those in winter. Originally, the production of melanin is a kind of biological reaction to protect the body from damage to the skin caused by ultraviolet (UV) rays increased in summer^21,22^. The use of the colorimeter (Mexameter) allowed useful assessment of the change in visual skin pigmentation. Between the winter and the summer, transition of dorsal pigmentation was well reflected in the changes in melanin values. These findings revealed that the dorsal skin of Capybaras had a protective effects on UV exposure during the summer.

Skin erythema values were also significantly lower in winter than in summer. The erythema values indicate the concentration of hemoglobin blood circulation at the measurement site. The application of this measurement tool permitted us to detect redness components of skin color. The elevated erythema values indicated that the environment in the summer promoted good skin microcirculation.

In this study, skin sebum was not detected in the normal Capybaras. In humans and other animals such as dogs and cats, skin sebum is constantly secreted from the sebaceous glands onto the epidermis. In contrast, Capybaras produced little sebum on the skin of the trunk body. Sebum has a role in preventing water evaporation from the skin and in maintaining the skin moisture. A very small amount of sebum involved considerable risk of developing dry and rough skin in the winter season. These results confirmed that the skin of Capybaras could not adequately adapt itself to the climate change in the winter.

Histopathological examination revealed several characteristics of the skin structure in Capybaras. First, Capybaras had a relatively thick epidermis. The thickness of the epidermis and the number of epidermal layers were consistent with those described in a previous report^23^. Animals coated with fine and dense hairs have considerably thin epidermis. The epidermis of cats and dogs is composed of 2–3 layers^24^, and the epidermis of guinea pigs and mice is also 3 or less layers^25^. It was probable that the thickness of the epidermis was associated with the population of hairs. The second feature was that melanin granules were found only on the dorsal skin. The epidermal melanocytes including abundant melanin granules were reflected in melanin values indicating skin pigmentation. Cutaneous melanin pigment plays a critical role in protection against harmful effects of solar radiation^26^. Epidermal melanin has important evolutionary and physiological implications, particularly for unclothed humans^26^. In contrast, many furred mammals lack melanogenically active melanocytes in their epidermis^26^. Capybaras seemed to acquire the ability to protect their skin from severe UV rays. Bathing for 21 consecutive days macroscopically provided noticeable improvement in the rough skin. The appearance and texture of the skin returned to the skin condition observed in the summer season. It has been reported in humans that continuous hot spring bathing improved the appearance of rough skin^27^.It should be noted that the aesthetic recovery from rough skin was related to the skin turnover. In addition, Capybaras recovered the glossy appearance on their hair. Capybaras spend a long time portion of their life in water and semiaquatic Capybaras naturally have hydrophobic hair surfaces covered with wax^28^. Hot spring bathing likely brought their hair into the best condition of hydrophobicity.

Daily bathing in alkaline hot spring brought prominent improvements to moisture content in the rough skin. 21 days after the start of the test, the skin moisture content was comparable to that of the skin in the summer season. It has been reported in humans that bathing in the alkaline hot water increases skin moisture content^29^. This finding implied that improvement in skin moisture was also associated with the advanced skin turnover.

In this experiment, the skin pH on the rough skin has not yet attained to the weakly alkaline condition, 21 days after the bathing procedure. In the light of improved the skin moisture content, continuous bathing is expected to bring increased skin pH close to the normal skin conditions found in the summer season.

The hot spring bathing test resulted in a significant decrease in skin melanin values. Bathing in alkaline hot spring has a peeling effect on the skin. Daily bathing with alkaline hot spring water gradually removed excessive melanin granules from the keratinous layer, resulting in a decrease in melanin values. It is known that depigmentation disorder (vitiligo) is alleviated by balneotherapy and spa therapy^30,31^. On the other hands, it has previously reported that hot spring water suppresses skin melanogenesis^32,33^. It is likely that hot spring bathing has some regulatory effects on the production of melanin granules.

21 days after bathing in hot spring, erythema values were elevated to those determined in the summer season. It has been reported in humans that hot bathing increases the cutaneous blood flow in the short term^34,35^. Our result is not fully explained by the short term thermal effect following bathing in hot spring. The result of this study suggested that successive hot spring bathing should provide normalization of skin condition and cutaneous microcirculation in the long term.

The evaluation of facial expression revealed that bathing in hot spring was comfortable for Capybaras. Ear position is widely used to assess the positive emotions of various animals^36–40^. It has been reported that rats, classified into the same the order Rodentia as Capybaras, also tend to reflect positive emotions in their ears, but there was no significant change in their eyes^41^. The results of our study showed that the eye score was useful for Capybaras as an indicator of comfortable status, though there were no significant changes in ear position.

The thermography revealed a heat retention effect of the body temperature after hot spring bathing. These results, in particular, taken from the extremities demonstrated the potential for preventing the body from getting too cold after hot spring bathing. It is known that hot spring water containing sodium chloride has features of heat-retention after bathing^42–45^. A previous study describes that Capybaras have a very small number of sweat glands^23^. Because the present study also showed that Capybaras had no eccrine sweat glands on the body surface, the sweat glands do not participate in thermal sweating. The hot spring water used in this study contained several salts such as sodium chloride in solution. It is probable that these salts cover the body surface with the fine membrane, and then the salts bound to the epidermal protein prevent the body heat from dissipating, as described in previous papers^42,45,46^. We are now in the process of investigating this problem. The skin is said to be the largest body’s organ at the interface between external and internal environment. The skin has the capability to differentially react to changes in external environment. The skin is continuously exposed to external environment factors (UV radiation and thermal changes). The skin barrier need to well adapt to optimal mechanism to protect, restore or maintain local and global homeostasis in relation to hostile environment. In recent advances, it has been proposed that precise coordination and execution of this responses should be mediated by a cutaneous neuroendocrine system^47^. This system is also able to reset the body homeostatic adaptation mechanisms^48^. From skin appearance, skin properties, comfortable status and thermogram, dermatological improvement strongly supports regulation of local and global homeostasis by skin neuroendocrine system, in the hot spring bathing experiment with Capybaras.

In conclusion, our results reveal that consecutive bathing in alkaline hot spring is highly effective in improving the rough skin. In addition, the present study documents the heat retention effect after bathing in hot spring. This experiment demonstrates that hot spring had significantly dermatological effect on the basis of evaluation for the skin in Capybaras. These findings provide potential that leads to the clue to the improvable effects of the hot spring bathing on human skin.

## Methods

### Analysis of hot spring water

Hot spring water supplied by Yuda hot spring distribution cooperative used in this study. Yuda hot spring is located in Yamaguchi City, Yamaguchi Prefecture, Japan. Yuda hot spring is categorized into non-volcanic hot springs which are not derived from volcanic activity. The temperature of the welling-up hot spring is as high as 72–76 $^\circ$C to the extent that the geothermal gradient alone cannot explain. Components of hot spring were analyzed by Yamaguchi Prefectural Institute of Public Health and Environment (Yamaguchi, Japan).

### Animals

Nine 2–12 years-old Capybaras (4 males and 5 females) were used in this series of studies. These animals were bred in Akiyoshidai Zoological Park Safari Land. The Capybaras were housed in groups in an animal room maintained at 24.2 to 27.9 $^\circ$C. By day, they were maintained in outdoors runs. They were fed timothy hay, cabbage, carrot and pellets for Herbivore. Water troughs were provided to allow ready access for all animals.

### Assessment of skin properties

Their skin conditions were investigated in the summer season (high temperature and high humidity) and in the winter season (low temperature and low humidity). The normal skin properties in summer was considered to be a reference value for the rough skin in winter. The items and measuring probes were as follows:

1. Moisture (Corneometer CM825; Courage + Khazaka Electronic GmbH, Cologne, Germany).
2. pH (Skin-pH-Meter PH905; Courage + Khazaka Electronic GmbH, Cologne, Germany).
3. Sebum (Sebmeter SM815; Courage + Khazaka Electronic GmbH, Cologne, Germany).
4. Melanin (Mexameter MX18; Courage + Khazaka Electronic GmbH, Cologne, Germany).
5. Erythema (Mexameter MX18; Courage + Khazaka Electronic GmbH, Cologne, Germany).

The former probes (1, 2 and 3) were connected with the measurement apparatus (Derma Unit SSC3; Courage + Khazaka Electronic GmbH, Cologne, Germany). The latter probes (4 and 5) were connected with another apparatus (Multi Display Devices MDD4; Courage + Khazaka Electronic GmbH, Cologne, Germany). Each probe was applied to the skin surface, touched the skin, and then a measured value was calculated and recorded on the apparatuses.

The measurement principle of each probe and the interpretation of the measured values are briefly described below.

Moisture (Corneometer CM825): Electrolysis is generated on the skin through the glass plate and electrostatic capacity is measured. Depending on the electrostatic capacity, skin moisture content is displayed on the apparatus as a relative value between 0 and 120 (dimensionless number). The high value means abundant skin moisture.

pH (Skin-pH-Meter PH905): The pH value is measured by the general glass electrode method using the potential difference between the glass electrode and the reference electrode.

Sebum (Sebmeter SM815): The amount of sebum is measured by calculating the light transmission of the tape that has absorbed the sebum secreted from the surface of the skin. The value range is 0 to 300 and the unit is µg/cm^2^.

Melanin and erythema (Mexameter MX18): The probe irradiates three different wavelength light to the measurement site, and the apparatus quantifies the density of melanin and hemoglobin (erythema) from the reflected light. Each value is shown as a relative value in the range 0–999 (dimensionless number), and the higher the value, the stronger the blackness and redness of the skin.

### Histological examination

Skin tissue specimens were obtained from both of the dorsal and abdominal sites under general anesthesia with medetomidine (50 µg/kg, Domitol, Nippon Zenyaku Kogyo Co., Ltd., Kohriyama, Japan), midazolam (0.15 mg/kg, Midazolam injection 0.5% [F], Fuji Pharma Co., Ltd., Tokyo, Japan) and Ketamine (1.5 mg/kg, Ketamin injection 5% [Fujita], Fujita Pharmaceutical Co., Ltd., Tokyo, Japan) combination(i.m.). These samples were taken from another Capybaras different from the animals used in this assessment of skin properties. This skin sampled from the individual suffering from injury served to examine the morphological structure. The collected samples were normal tissue without injury. The tissue specimens were fixed in 10% neutral buffered formalin and 4-µm paraffin sections were stained with hematoxylin and eosine (HE) and by Fontana-Masson (FM) method, van Gieson’s (vG) method and Weigert’s (WG) method.

### Experiment schedule of hot spring bathing

**Figure 6:**
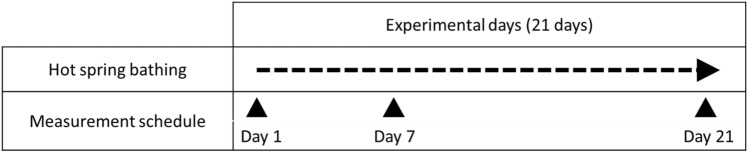
Experimental schedule of hot spring bathing. Capybaras took a bath every day for at least 30 min. The measurements were taken before bathing in the morning of the day.

A series of experiments were performed in the Akiyoshidai Zoological Safari Land during winter. The experiment schedule for bathing is shown in Fig. 6. Before the start of the bathing experiment, it was observed that the Capybaras had rough skin. After that, the animals took a hot spring bath every day for at least 30 min over 21 days. Skin condition was measured on days 1, 7, and 21 of the test, and the measurements were taken before bathing in the morning of the day. The measured value on day 1 reflects the rough skin condition before the bathing test, which is the baseline for the bathing test. The hot spring temperature was adjusted about 35 $^\circ$C.

### Evaluation of comfortable status

The facial expression of Capybaras was scored to evaluate comfortable status during bathing in hot spring. Capybaras display stereotypical changes mainly in the site of eyes and ears. In this study, their comfortable status was evaluated by scoring photographs taken from a variety of environmental conditions. The comfortable status of each part was classified into 3 degrees: “0” = calm at baseline, “1” = moderate comfortable and “2” = obvious comfortable. Eye score was evaluated based on the eye shape, and the criteria were defined as follows: “0” = completely open, “1” = half open and “2” = almost closed. By ear position, ear score evaluated as follows: “0” = facing forward, “1” = slightly pulled back and “2” = pulled back exaggeratedly and facing side.

### Skin thermography

The body surface temperature of the animals was contactlessly recorded with thermograph FLIR E4 (FLIR Systems, Inc., Oregon, USA). Thermograms were visually measured immediately before bathing and 0, 10, 20 and 30 min after bathing. (Thermographs were taken immediately before bathing and 0, 10, 20, 30 min after bathing.) The surface temperature was measured in 3 areas (head, trunk body and the distal portions of extremities). These image data for each area were analyzed using FLIR Tools (FLIR Systems, Inc., Oregon, USA) and the changes in skin temperature were calculated by mean ± SD.

### Data and statistical analysis

All statistical analysis were performed by Statcel 4 (OMS Publishing Inc., Saitama, Japan) and the significant difference was determined by Student’s t-test, Wilcoxon rank sum test and Dunnett's test which is one of the multiple comparison methods (p < 0.05 and p < 0.01). Spearman's rank correlation coefficient test was applied to the calculation of the correlation. (p < 0.05).

### Ethical statement

All procedures involving animals were approved by the Institutional Animal Care and Use Committee of Yamaguchi University (approval No. 284: April 13, 2018) and followed the Guidelines of Animal Care and Experiments of Yamaguchi University. The animal care and use program for Advanced Research Center for Laboratory Animal Science in Yamaguchi University has been accredited by AAALAC International since 2018. This experiment was performed in accordance with ARRIVE guidelines.
